# Dataset on volatile compounds in susceptible and resistant chili variety to fruit fly infestation

**DOI:** 10.1016/j.dib.2018.12.018

**Published:** 2018-12-13

**Authors:** Rinda Kirana, Asih Kartasih Karyadi, Ahmad Faizal, Tati Suryati Syamsudin

**Affiliations:** a School of Life Sciences and Technology, Institut Teknologi Bandung, Ganesa 10, Bandung 40132, Indonesia; b Indonesian Vegetable Research Institute (IVEGRI), Tangkuban Parahu 517, Bandung Barat 40391, Indonesia

## Abstract

This data article describes the analysis of volatile compounds in susceptible and resistant chili variety via gas chromatography/mass spectrometry (GC–MS). The volatile compounds from two chili varieties which have different level of resistance to fruit fly infestation were captured by a solid-phase micro extraction method. The obtained extracts were then subjected to GC–MS for separation and identification of the compounds in three developmental stages (buds, flowers and fruits). The retention times and the detected compounds were identified as well as their relative peak areas. The data were provided in Supplementary 1 (Table 1) and Supplementary 2 (Table 2).

**Specifications table**

**Table:** 

Subject area	Agricultural and Biological Sciences
More specific subject area	Plant Resistance
Type of data	Microsoft Excel Worksheet
How data were acquired	Volatile compound from two chili variety (buds, flowers and fruits) were analyzed using gas chromatography (GC: 7890A, Agilent Technologies, Inc.) coupled with mass spectrometry (MS: 5975C, Agilent Technologies, Inc.).
Data format	Analyzed
Experimental factors	Two varieties of chili (susceptible and resistant to fruit fly infestation); three developmental stages of chili (buds, flowers and fruits).
Experimental features	Determination of retention times; identification of volatile compounds and their relative peak area from the GC chromatogram.
Data source location	West Java, Indonesia
Data accessibility	The data are available with this article and accessible to the public.

**Value of the data**

• The data showed the difference volatile profile from susceptible and resistant chili to fruit fly infestation.
• Data could be used by other researchers for understanding defense mechanism of chili to fruit fly infestation.
• Data could be used by breeders on breeding program for chili resistance to fruit fly infestation.

## 1 Data

The Microsoft Excel Worksheet that is provided as supplementary data for this article (Supplementary 1 and 2). The data contain the retention times, volatile compound names and the relative peak area (in percentage) of the buds, flowers and fruits from two chili varieties (susceptible and resistant variety).

## 2 Experimental design, materials, and methods

Chili (*Capsicum annuum* L.) from two variety (susceptible and resistant to fruit fly infestation) planted in the screen house of Indonesian Vegetable Research Institute (IVEGRI) in Lembang, West Java Indonesia and harvested during three stages (buds, flowers and fruits). The chili plants were grown following the technique usually implemented by IVEGRI. Volatile compounds analysis conducted in flavour laboratory, Indonesian Centre for Rice Research (ICRR) in Subang, West Java, Indonesia. The compounds were extracted using the protocol described by Wahyuni et al. [1], Junior et al. [2] and Aluja et al. [3] with some modifications.

The samples were extracted using SPME fiber coated with 50/30 µm divinylbenzene/carboxen/polymethylsiloxane (DVB/CAR/PDMS) (Supelco Co., Bellefonte, PA, USA). Ten grams of buds, flowers and fruits were placed in 40 mL SPME glass vial with PTFE/silicone septa and incubated at 30 °C for 30 min. The SPME fiber was injected into a gas chromatograph at 250 °C for 5 min in splitless mode. The oven temperature was initially at 45 °C held for 2 min and then increased to 250 °C at the rate of 5 °C/min for 5 min. The volatile compounds were identified based on their retention times in gas chromatograph Agilent 7890A equipped with mass spectrometer Agilent 5975C. DB-5 MS column was used for the separation. Gas carrier was helium 1 ml/min. The relative amounts of volatile compounds in each part from two chili varieties were determined by comparing spectra of each compound with library NIST08.
